# Cytological and transcriptome analyses reveal abrupt gene expression for meiosis and saccharide metabolisms that associated with pollen abortion in autotetraploid rice

**DOI:** 10.1007/s00438-018-1471-0

**Published:** 2018-07-04

**Authors:** Lin Chen, Muhammad Qasim Shahid, Jinwen Wu, Zhixiong Chen, Lan Wang, Xiangdong Liu

**Affiliations:** 1 State Key Laboratory for Conservation and Utilization of Subtropical Agro-Bioresources, South China Agricultural University, Guangzhou, 510642 China; 2 Guangdong Provincial Key Laboratory of Plant Molecular Breeding, South China Agricultural University, Guangzhou, 510642 China

**Keywords:** Autotetraploid rice, Pollen development, Carbohydrate metabolism, Saccharides’ transporters, Meiosis

## Abstract

Autotetraploid rice is a useful germplasm that has four chromosome sets and strong biological advantages; however, low fertility limits its commercial utilization. Little information is available about the DNA variation and differential gene expressions associated with low fertility in autotetraploid rice. In the present study, 81 SNPs and 182 InDels were identified in T449 (an autotetraploid rice line with low fertility) compared to E249 (diploid counterpart) by whole-genome re-sequencing. We detected only three non-synonymous SNPs and six large-effect InDels, which were associated with three and six genes, respectively. A total of 75 meiosis-related differentially expressed genes were detected during the meiosis stage by transcriptome analysis, including *OsMTOPVIB*, which is essential for meiotic DSB formation, and *OsMOF*, which takes part in homologous chromosome pairing and synapsis. Approximately 20.69% lagging chromosome at metaphase I and 4.65% abnormal tetrad were observed in T449. Moreover, transcriptome analysis revealed down-regulation of a sucrose transporter (*OsSUT5*) and two monosaccharide transporters (*OsMST1* and *OsMST8*) in T449 at the single microspore stage, and their expression levels were verified by qRT-PCR. Cytological observation of saccharide distribution showed abnormal accumulation of saccharides in T449 and the contents of fructose and glucose were markedly higher in T449 than E249 at the single microspore stage. Our results suggested that polyploidy not only induces abrupt expression changes in the meiosis-related genes that lead to abnormal chromosome behavior, but also causes changes in the saccharide distribution and expression patterns of saccharide-related genes, which jointly causes sterility in the autotetraploid rice.

**Electronic supplementary material:**

The online version of this article (10.1007/s00438-018-1471-0) contains supplementary material, which is available to authorized users.

## Introduction

Whole-genome duplication (WGD) or polyploidy has played an important role in plant evolution. Environmental change or stress increased the short-term adaptive potential of polyploids due to changes in gene expression patterns and increased genetic variation (Wendel 2000; Soltis et al. 2009; Van de Peer et al. 2017). There are two major types of polyploids in plants: allopolyploid and autopolyploid. Allopolyploids originate through the duplication of chromosome sets of different species, a major pathway for plant evolution, which may have a survival advantage, because different chromosome sets are key determinants of adaptive success in the new environment (Soltis et al. 2009; Paun et al. 2011; Van de Peer et al. 2017). Autopolyploids involve whole-genome duplication within-species, which is more prevalent than indicated by taxonomy alone and has become an important element of plant diversity (Soltis et al. 2007; Van de Peer et al. 2017). Autopolyploid species are widely found in plants and have higher economic and resistance importance than their progenitors (Gebhardt and Valkonen 2001; Barker et al. 2016; Van de Peer et al. 2017). Autotetraploid rice has strong biological advantages over diploid rice, such as huge vegetative organs, high yield potential, and wide adaptability (Shahid et al. 2011, 2012; Tu et al. 2014; Yang et al. 2014). However, low fertility is a major obstacle in the utilization of autotetraploid rice (He et al. 2011a; Shahid et al. 2013a; Wu et al. 2015; Li et al. 2017). To improve the fertility and yield of autotetraploid rice, it is of utmost importance to investigate pollen development and its molecular mechanism in this organism.

Partial pollen sterility is a main reason for low fertility in autotetraploid rice (Shahid et al. 2010; Li et al. 2016). The previous studies have shown that abnormal chromosome behavior and abnormal microtubule organization are the main cause of pollen abortion in autotetraploid rice (He et al. 2011a, b; Wu et al. 2014). Transcriptome analysis has suggested that polyploidy enhanced multi-allelic interactions at pollen sterility loci and increased chromosomal abnormalities in autotetraploid rice (Wu et al. 2015). In addition, small RNA sequencing indicated that the partial sterilities of pollen and embryo sac were caused by specific differentially expressed miRNAs in autotetraploid rice (Li et al. 2016, 2017). Two photoperiod- and thermo-sensitive genic male sterile lines (PS006 and PS012) of polyploid rice were developed and their hybrids showed great heterosis and enormous potential for the improvement of rice quality and productivity (Zhang et al. 2017).

Next-generation sequencing is widely used in genome sequencing, transcriptome sequencing, small RNA sequencing, chromatin immunoprecipitation sequencing (ChIP-Seq), and DNA methylation sequencing. Genomic re-sequence technology is an efficient way to detect genetic variations (Han and Huang 2013), which play an importance role in the QTL mapping, marker-assisted genomic selection, and haploid-type analysis (McCouch et al. 2010). Genome-wide association studies (GWAS) have concentrated on the analysis of genes associated with heading date and grain-related traits using whole-genome re-sequencing (Huang et al. 2012). Fu et al. (2016) identified numerous SNPs and InDels in two *indica* rice (RGD-7S and Tai Feng B) and obtained DNA polymorphic markers by re-sequencing two rice cultivars. RNA transcript technology (RNA-seq) is a helpful tool for understanding of differentially expressed genes and the expression patterns of stage-specific genes in plants. RNA-seq has been applied to detect differentially expressed genes under abiotic stresses in diploid rice (Jin et al. 2013; Fu et al. 2017). In addition, RNA-seq has also been applied to identify differentially expressed genes in autotetraploid rice compared to diploid rice during pollen development (Wu et al. 2014). Guo et al. (2017) detected many genes associated with fertility and heterosis in neo-tetraploid rice hybrids by RNA-seq. However, little information about genome-wide DNA variation combined with RNA-seq is available in autotetraploid compared to diploid rice.

To detect the genomic variations between autotetraploid rice line (T449) and its diploid rice counterpart (E249), which is a famous diploid rice variety harbouring double-neutral genes at the *Sa* and *Sb* pollen sterility loci and the *S*_*5*_^*n*^ gene that can overcome *indica–japonica* hybrid sterility (Shahid et al. 2013b) was re-sequenced using a high-resolution technique. T449 had been self-crossed for more than 25 generations in our lab, and has stable agronomic traits. Moreover, chromosome behavior and histological observations were used to investigate pollen development in T449 and E249. Transcriptome analysis of anther development was also performed during meiosis and single microspore stages to detect differentially expressed genes (DEGs) between T449 and E249. This study was planned (1) to observe chromosome behavior and saccharide distribution in autotetraploid rice during pollen development and (2) to detect genes that might be associated with low pollen fertility in autotetraploid rice. We found that abrupt changes in the expression patterns of meiosis and saccharide-related genes cause low fertility in autotetraploid rice.

## Materials and methods

### Rice material

An autotetraploid rice line (T449) and its diploid counterpart (E249) were used in this study. T449 was developed from E249 by treatment with colchicine and self-crossed for more than 25 generations at our farm. All materials were planted at the experimental farm of South China Agricultural University (SCAU) under natural conditions, and management practices followed the recommendations for the area.

### Observation of chromosome behaviour and pollen fertility

Chromosome behaviour was observed according to Wu et al. (2014). The spikelets of T449 and E249 were harvested from the shoots of rice plants with − 2 to 4 cm between their flag leaf cushion and the second to last leaf cushion, and fixed in Carnoy’s solution (ethanol:acetic acid = 3:1) for 24 h. The samples were stored in 70% ethanol at 4 °C after washing. Anthers were dissected from the floret using a dissecting needle and forceps, and were squashed and placed in a small drop of 1% acetocarmine on a glass slide. After 1–2 min, the glass slide was covered with a slide cover and observed under a microscope (Motic BA200).

Pollen fertility was estimated by the ratio of normal and abnormal pollen grains, which were observed by staining with 1% I_2_–KI under a microscope (Motic BA200). Normal pollens are fully stained, while abnormal pollens can be divided into two types based on staining and pollen morphology, including stained abortive pollens and empty abortive pollens. Stained abortive pollens are smaller than normal pollens or not fully stained and empty abortive pollens are smaller than normal pollens, and are empty or colourless (Zhang and Lu 1989).

### Histological observation

The anthers of T449 and E249 were harvested, fixed, dehydrated, and embedded in a Leica 7022 Histeresin Embedding Kit (7022LR) according to the manufacturers’ protocol (Heraeus Kulzer). Sections of 1–2 µm thickness were cut with the microtome (Leica RM2235), and were dried at 60 °C for 24 h. The sections were stained in 0.5% periodic acid (m/v) and with Schiff reagents [0.05% fuchsin basic (m/v), 0.05% sodium metabisulphite (m/v), and 10% HCl (v/v)], and finally with 0.05% toluidine blue (m/v) (Feder and OBrien 1968). The sections of anthers were observed and photographed using a microscope (Motic BA200).

### Determination of saccharides’ content in anther

The anthers of T449 and E249 at the meiotic and single microspore stages were collected to confirm the pollen development stages using DAPI fluorescence staining method (Table S1). The 500 mg samples of fresh anthers were air-dried at 110 °C in an oven for 15 min and kept at 65 °C for 12 h in the same oven. The 50 mg samples of dried anthers were extracted with 80% ethanol aqueous solution, and then placed in a hot water bath at 80 °C for 40 min. The samples were centrifuged at 4000 rpm for 10 min and supernatant was removed. The same process was repeated twice and the supernatants were collected. The activated carbon was added to the supernatant for decolorization at 80 °C for 10 min, and the volume was adjusted to 5 ml. Finally, the mixed supernatant was filtered and prepared to measure the contents of fructose, glucose, and sucrose using kits purchased from Suzhou Keming Bioengineer Company, China. All measurements were performed in triplicate.

### Whole-genome re-sequencing analysis

The genomic DNAs of E249 and T449 were extracted from young leaves using a modified CTAB method (Cota-Sanchez et al. 2006). The library was prepared according to the manufacturer’s protocol. The genomic re-sequencing was performed on the Illumina Hiseq 2500 platform (Biomarker Technologies, Beijing, China). The procedure was performed according to the standard Illumina protocol. The generated FASTQ file quality was evaluated using FastQC (http://www.bioinformatics.babraham.ac.uk/projects/fastqc/). The low-quality reads [reads with sequencing adapter, reads with more than 10% N content, and reads with more than 50% low-quality bases (< 10)] were removed, and the filtered high-quality reads were then mapped onto the Nipponbare reference genome using BWA software. The SNPs and InDels were detected by GATK software, and the base mutation of SNPs, physical location of the corresponding chromosome, the size of the small InDels, and positions on the corresponding chromosomes were detected. The copy number variations (CNVs) were identified using FREEC software, and the breakpoint threshold was 0.8 (window = 50,000), while other parameters were set as default. The SNPs and InDels were annotated using SnpEff software and CNVs were annotated based on the GFF file of the Nipponbare reference genome.

### Validation of SNPs and InDels

Based on the re-sequencing results, 38 SNPs and InDels were randomly selected for validation. Primer Premier 5.0 was employed to design oligonucleotide primers, with a product length that ranged from 400 to 800 bp (Table S2). The genomic DNAs of E249 and T449 were used as template to amplify DNA by the polymerase chain reaction (PCR). The PCR process was 94 °C for 5 min, followed by 35 cycles of 95 °C for 45 s, 55 °C for 45 s, and 72 °C for 1 min, and a final extension at 72 °C for 5 min. The PCR products were sequenced by Sanger sequencing, and the sequence alignments were performed using ClustalW software.

### RNA extraction and RNA-seq experiments

Total RNA was extracted from anthers at the meiotic and single microspore stages. All samples were collected in three biological replicates and stored at − 80 °C for RNA solation. The total RNA from each sample was extracted from the anthers according to the manual instructions of the TRIzol reagent (Life technologies, California, USA). The library was prepared according to the manufacturer’s protocol. The RNA-seq was performed on the Illumina HiSeq 2500 sequencing platform (Biomarker Technologies, Beijing, China). Using the Illumina paired-end RNA-seq approach, we sequenced the transcriptome that generated millions of paired-end reads. Low-quality reads (adaptor sequences, unknown nucleotides > 5%, or *Q*20 < 20% [percentage of sequences with sequencing error rates < 1%)] were removed. The clean reads, which were filtered from the raw reads, were mapped onto the Nipponbare reference genome using Bowtie2 and Tophat2 softwares with the default parameters. The aligned records from the aligners in the BAM/SAM format were further examined to remove potential duplications. Gene expression differences between samples were detected using the DESeq package (Anders and Huber 2010). The fragments per kilobase (FPKM of transcript per million fragments mapped) values were used to estimate gene expression levels (Trapnell et al. 2010). The false discovery rate (FDR) was used to determine the threshold of the *p* value in multiple tests. The genes were used for subsequent analysis using FDR ≤ 0.05 and the absolute value of log2 (fold change) ≥ 1.

### Statistical analysis of RNA-seq data

Hierarchical analysis was performed using Cluster 3.0 software after normalization. Venny software was used to identify the overlapped differentially expressed genes in different samples (http://bioinfogp.cnb.csic.es/tools/venny/). Transcription factor data were downloaded from the database DRTF (Jin et al. 2017). Gene ontology (GO) enrichment analysis was employed for functional categorization of differentially expressed genes using the Plant GeneSet Enrichment Analysis Toolkit (http://structuralbiology.cau.edu.cn/PlantGSEA/) and AgriGO tool (http://bioinfo.cau.edu.cn/agriGO/).

### Real-time qRT-PCR analysis

A total of 18 DEGs were selected for validation by qRT-PCR. The gene-specific primers were designed using Primer Premier 5.0 software (Table S3). Total RNA was taken from sequenced samples, and the first-strand cDNA was synthesized using the PrimeScript™ II first-strand cDNA synthesis kit (TaKaRa) according to the manufacturer’s instructions. The qRT-PCR was performed on the Lightcycler480 system (Roche) using the Prime Script RT reagent Kit with gDNA Eraser (TaKaRa). The qRT-PCR reaction condition was 30 s at 95 °C, with 40 cycles of 95 °C denaturation for 5 s and 60 °C annealing and extension for 20 s. The relative expression levels of genes were calculated using the 2^−ΔΔCt^ method (Livak and Schmittgen 2001). All qRT-PCR reactions were performed in triplicate.

### Data availability

All the data were submitted to NCBI Sequence Read Archive database under accession number PRJNA436888.

## Results

### Characterization of autotetraploid rice (T449)

**Fig. 1:**
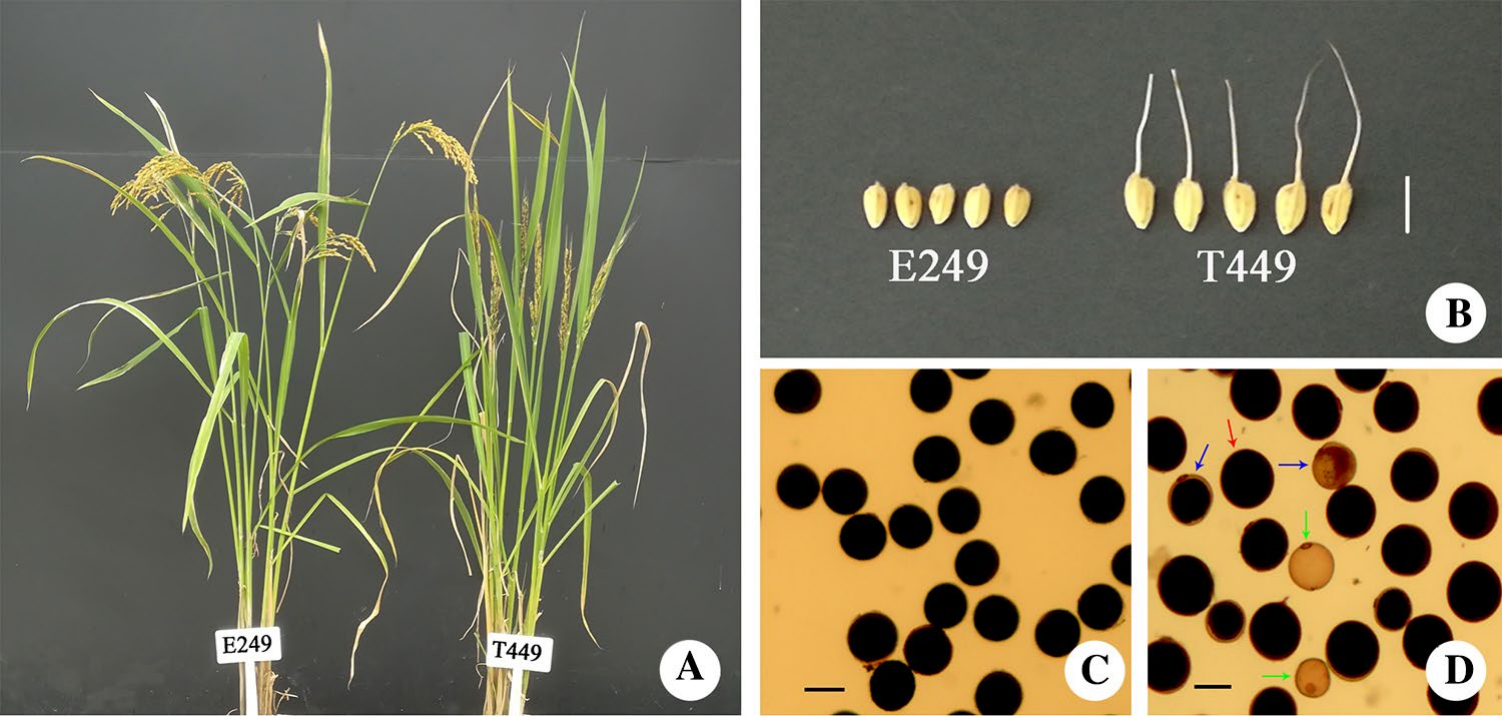
Plant type, grain shape, and pollen fertility of autotetraploid (T449) and diploid rice (E249). **a** Plant appearance of T449 and E249, **b** grains shape of T449 and E249, bar = 1 cm. Pollen fertility of E249 (**c**) and T449 (**d**) observed by staining with 1% I_2_–KI. Blue arrows indicate stained abortive pollen, green arrows indicate empty abortive pollen, and red arrows indicate normal pollen. Bar = 100 µm. (Color figure online)

Obvious differences were detected between T449 and E249 for the main agronomic traits (Fig. 1), including plant height, grain length, grain width, 1000-grain weight, and panicle length, but there were non-significant differences in flag leaf length and width (Table 1). Grain length, grain width, and 1000-grain weight were significantly larger, and plant height and number of effective panicles were significantly lower in T449 than in E249 (Table 1 and Fig. 1). In addition, stained abortive and empty abortive pollens were found in the T449 (Fig. 1), and pollen fertility and seed setting were 54.09 and 34.47% in T449, respectively, significantly lower than E249 (Table 1). These results showed that T449 had obvious biomass advantages over its diploid counterpart, and displayed significant differences in agronomic traits.

**Table 1:** Main agronomic traits and pollen fertility of autotetraploid rice (T449) and diploid rice (E249)

Traits	E249	T449
Plant height (cm)	96.5 ± 2.91	84.5 ± 4.52**
Flag leaf length (cm)	35.36 ± 5.83	36.49 ± 5.41
Flag leaf width (cm)	2.05 ± 0.06	2.04 ± 0.11
Grain length (mm)	7.19 ± 0.10	8.99 ± 0.37**
Grain width (mm)	3.05 ± 0.04	3.73 ± 0.04**
1000-grain weight (g)	18.27 ± 0.43	32.34 ± 1.37**
Panicle length (cm)	23.4 ± 1.24	25.33 ± 1.02**
Number of effective panicles	7.35 ± 1.27	4.25 ± 1.12**
Pollen fertility (%)	94.43 ± 1.72	54.09 ± 3.13**
Seed setting (%)	89.38 ± 2.33	34.47 ± 9.11**

**Significant difference at *p* < 0.01

### Chromosome behaviour during PMC meiosis in the autotetraploid rice

**Fig. 2:**
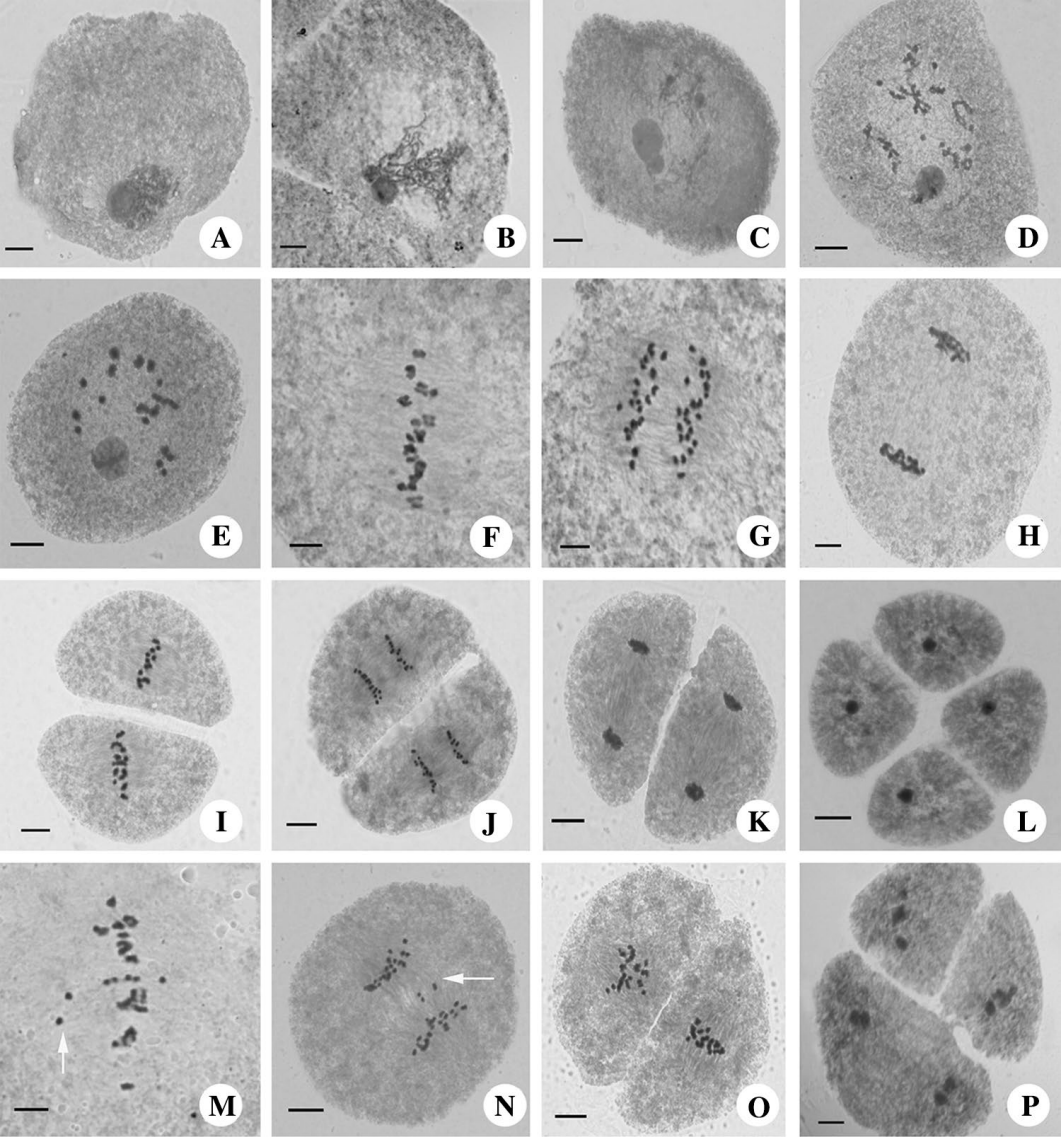
Chromosome behaviours during PMC meiosis in an autotetraploid rice line. **a** Zygotene, **b, c** pachytene, **d** diplotene, **e** diakinesis, **f** metaphase, **i, g** anaphase I, **h** telophase I, **i** metaphase II, **j** anaphase II, **k** telophase II, **l** tetrad stage, **m** abnormal metaphase I, **n** abnormal anaphase I, **o** abnormal metaphase II, **p** abnormal tetrad stage. Bar = 10 µm

Meiotic stages in T449 were consistent with E249, and could be divided into nine development stages, including prophase I (leptotene, zygotene, pachytene, diplotene, and diakinesis) (Fig. S1 A, B and Fig. 2a–e), metaphase I (Fig. S1C and Fig. 2f), anaphase I (Fig. S1D and Fig. 2g), telophase I (Fig. S1E and Fig. 2h), prophase II, metaphase II (Fig. S1F and Fig. 2i), anaphase II (Fig. S1G and Fig. 2j), telophase II (Fig. S1H and Fig. 2k), and tetrad (Fig. S1I and Fig. 2l). The bivalent was the most common chromosome configuration at diakinesis and metaphase I in E249, while tetravalent was most frequent in T449 at the same stages (Table 2). Many abnormal chromosome behaviours were observed in T449, including chromosome lagging at metaphase I (Fig. 2m), chromosome straggling at anaphase I (Fig. 2n), abnormal spindle at metaphase II (Fig. 2o), and asynchronous cell division at the triad stage (Fig. 2p). During meiosis I, the average frequencies of chromosomal abnormalities were 40.36, 20.69, and 2.62% higher in T449 than E249 at metaphase I, anaphase I, and telophase I, respectively. In meiosis II, the average abnormal frequencies were 31.62, 56.03, 7.96, and 4.65% higher in T449 than E249 at metaphase II, anaphase II, telophase II, and tetrad, respectively (Table 3).

**Table 2:** Meiotic chromosome configurations of autotetraploid (T449) and diploid (E249) rice

	Number of cells	Stages	Chromosome configuration
E249	160	Diakinesis	(0.2 ± 0.64)I + (11.9 ± 0.32)II
	159	Metaphase I	(0.16 ± 0.55)I + (11.92 ± 0.27)II
T449	140	Diakinesis	(0.91 ± 1.39)I + (5.85 ± 3.39)II + (0.18 ± 0.55)III + (8.71 ± 1.88)IV
	142	Metaphase I	(1.29 ± 1.6)I + (6.47 ± 3.25)II + (0.2 ± 0.58)III + (8.29 ± 1.57)IV

I, II, III, and IV represent univalent, bivalent, trivalent, and tetravalent, respectively

**Table 3:** Frequency of abnormal chromosome behaviours during meiosis in autotetraploid rice (T449) and its diploid (E249) counterpart

Stage	E249		T449	
	Number of cells	Abnormal (%)	Number of cells	Abnormal (%)
Metaphase I	244	2.05	275	40.36
Anaphase I	220	3.18	232	20.69
Telophase I	224	0.45	229	2.62
Metaphase II	256	3.52	253	31.62
Anaphase II	181	7.18	141	56.03
Telophase II	259	4.63	289	7.96
Tetrad stage	249	1.20	258	4.65

### Whole-genome re-sequencing of autotetraploid and diploid rice

A total of 107131936 and 100395344 clean reads were obtained in E249 and T449 using re-sequencing, and filtered from 107605398 and 100940422 raw reads, respectively. The G/C contents were 44.48 and 45.1% in E249 and T449, respectively. Approximately 98.34% (E249) and 98.13% (T449) of clean reads were mapped onto the Nipponbare reference genome, respectively. Moreover, 94.96 and 94.44% of these reads were uniquely mapped, and 94.32 and 93.88% were found at the 10× coverage depths, and the reads’ coverage depth were 37× and 35× in E249 and T449, respectively (Table 4).

**Table 4:** Summary of general sequencing data of autotetraploid (T449) and diploid (E249) rice mapped onto Nipponbare reference genome

	E249	T449
Clean bases	16049400548	15040007266
Raw reads	107605398	100940422
Clean reads	107131936	100395344
Percent of bases ≥ *Q*30 (%)	87.31	86.81
Mapped (%)	98.34	98.13
Properly mapped (%)	94.96	94.44
Coverage ratio 10× (%)	94.32	93.88
GC content (%)	44.48	45.1
Average coverage depth	37	35

To understand the genomic variations between E249 and T449, the DNA polymorphisms were further analyzed. The two filter conditions (coverage ≥ 10 and ≤ 100, and removal of heterozygous SNPs and InDels) were applied to minimize the detection of false-positive SNPs and InDels between E249 and T449. A total of 81 SNPs and 182 InDels were identified in T449 compared to E249 (Table S4). To validate using Sanger sequencing, we randomly selected 38 variation sites of SNPs and InDels from E249/T449 after PCR amplification. The DNA sequences were consistent with the re-sequencing data, suggesting that the re-sequencing data are reliable. Only three non-synonymous SNPs and six large-effect InDels were detected, which were associated with three and six genes, respectively, including cysteine protease family gene (*Os04g0402300*), methyltransferase (*Os09g0415700*), pectinesterase (*Os01g0634600*), disease-resistance gene (*Os11g0224900*), verticillium wilt disease-resistance gene (*Os01g0158600*), two NBS-LRR-type disease-resistance genes (*Os11g0597700* and *Os11g0686500*), and two expressed genes (*Os08g0528700* and *Os10g0134033*, Table S4). However, there was no variation at the pollen sterility loci *Sa* and *Sb*, and *S*_*5*_ gene between T449 and E249. These results suggested that the reason for the low pollen fertility of T449 may not be SNP and InDel variations.

A total of 60 CNVs were detected between E249 and T449, which were associated with 617 genes. GO-enrichment analysis of the 617 genes revealed that 13 GO terms were significantly enriched in the biological process category, and only a membrane (GO:0016020) term was detected in the cell component category (Table S5). Five GO terms were detected in the molecular function category, including transferase activity (GO:0016740), kinase activity (GO:001630), transferase activity, transferring phosphorus-containing groups (GO:0016772), catalytic activity (GO:0003824), and lipid binding (GO:0008289). Furthermore, we compared the genes related to CNVs in T449 with the data reported for rice anther meiosis-stage-related genes (Fujita et al. 2010; Yant et al. 2013; Wright et al. 2015). We identified two meiosis-related genes from these comparisons, including *OsAM1* (*Os03g0650400*), which plays a fundamental role in building the proper chromosome structure at the beginning of meiosis (Che et al. 2011) and *RAD17* (*Os03g0242100*), which regulates DNA damage repair and homologous recombination (Heitzeberg et al. 2004). However, the expression patterns of two meiosis-related genes displayed non-significant changes between E249 and T449 according to qRT-PCR results (Fig. S2).

### Differentially expressed genes in autotetraploid and diploid rice

Meiosis and single microspore stages play important roles in determining the pollen fertility of rice. Therefore, RNA-seq was employed to study the global gene expression of the two stages in T449 compared to E249. In total, approximately 230 million clean reads were detected in anthers at the meiosis and single microspore stages. The clean reads were aligned against the Nipponbare reference genome, and 84.28–89.73% annotated transcripts of the reference genome were obtained in T449 and E249, respectively (Table S6). All of the correlation coefficients were higher than 0.85 among the three biological replications (Table S7), and principal component analysis (PCA) showed that replicate samples of each development stage clustered together (Fig. S3), which suggests that expression patterns have high similarity between biological replications. A total of 18 genes were selected to confirm RNA-seq data using qRT-PCR, including 11 and 7 genes at the meiotic and single microspore stages, respectively. The results were consistent with the RNA-seq data (Fig. S4), suggesting that the RNA-seq data are reliable.

**Fig. 3:**
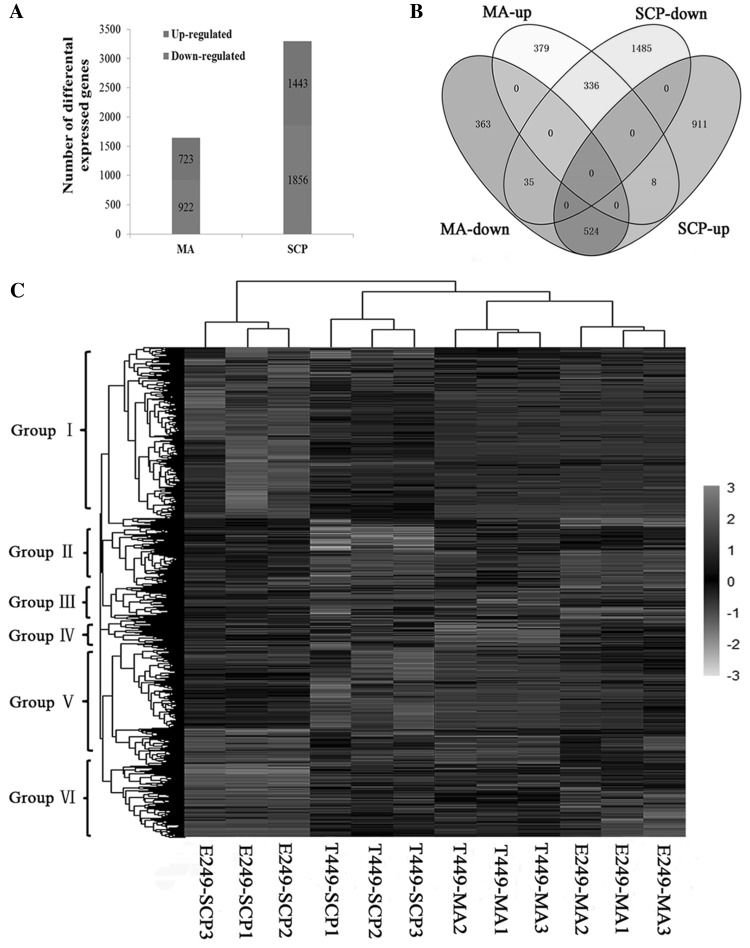
Differentially expressed genes (DEGs) in autotetraploid rice compared to diploid rice at meiotic and single microspore stages. **a** Number of up- and down-regulated DEGs in autotetraploid rice compared to diploid counterpart during both stages. **b** Venn diagram of DEGs in autotetraploid rice compared to diploid rice during both stages. **c** Expression patterns of DEGs between autotetraploid rice and its diploid counterpart in both stages. MA and SCP represent meiosis and single microspore stages, respectively. MA-up and MA-down represent up-regulated and down-regulated genes in autotetraploid rice compared to diploid rice during meiosis stage, respectively. SCP-up and SCP-down represent up-regulated and down-regulated genes in autotetraploid rice compared to diploid rice during single microspore stage, respectively

In total, 4944 genes displayed differential expression patterns, including 1645 and 3299 genes at the meiosis and single microspore stages in T449 compared to E249, respectively (Fig. 3a, Tables S8 and S9). Of these DEGs, 524 genes were found to be down-regulated during the meiosis stage, but up-regulated at the single microspore stage, while 336 genes were up-regulated at the meiosis stage, but down-regulated at the single microspore stage (Fig. 3b). In a comparison of the meiosis and single microspore stages, 7968 and 4659 DEGs were detected in E249 and T449, respectively. Of these DEGs, 1032 down-regulated and 1376 up-regulated genes were common in E249 and T449 (Fig. S5A). These results showed that many genes were expressed from meiosis to single microspore stages in different ploidy rice. Comparative analysis of the meiosis and single microspore stages revealed that 280 genes were up-regulated in E249, but down-regulated in T449, and 229 genes were down-regulated in E249, but up-regulated in T449 (Fig. S5A). Some genes were specifically co-expressed in different ploidy levels and pollen development stages (Fig. S5B). These results showed that genes were transcriptionally active during the meiosis and single microspore stages in different ploidy rice. Of these 4944 DEGs, which could be divided into six groups (Fig. 3c), 160 DEGs belonged to 36 families encoding transcription factors (TFs), including 73 and 133 DEGs expressed during the meiosis and single microspore stages, respectively (Fig. S6). The NAC families were most abundant in both stages, including 12 and 16 NAC TFs at the meiotic and single microspore stages, respectively. Among them, 27 and 86 TFs were specifically expressed at the meiotic and single microspore stages, respectively. Four TFs (GATA, GRAS, MYB, and MYB-related) were significantly down-regulated, and three TFs (C3H, CO-like, and MIKC) were found up-regulated during both development stages (Table S10).

Gene ontology (GO) enrichment analysis of the 4944 DEGs showed that a total of 29 and 37 GO terms were significantly enriched at the meiosis and single microspore stages in T449 compared to E249, respectively. In the biological processes category, GO terms related to the generation of precursor metabolites and energy (GO:0006091), response to abiotic stimulus (GO:0009628), response to biotic stimulus (GO:0009607), catabolic process (GO:0009056), and carbohydrate metabolic process (GO:0005975) were detected during both development stages. In molecular function category, catalytic activity (GO:0003824) and transporter activity (GO:0005215) were enriched in both stages. A total of 11 GO terms, such as plastid (GO:0009536), intracellular membrane-bounded organelle (GO:0043231), membrane-bounded organelle (GO:0043227), intracellular organelle (GO:0043229), and organelle (GO:0043226), were identified in the cell component category during both development stages (Figs. S7, S8, and S9).

KEGG pathways analysis showed that 237 of 1645 DEGs at the meiosis stage and 444 of 3299 DEGs at the single microspore stage were enriched in 26 functional terms in T449 compared to E249. Interestingly, 61 and 101 DEGs were involved in the carbohydrate metabolism categories during the meiosis and single microspore stages, respectively (Fig. S10). The aforementioned DEGs were enriched in 117 subcategories, and 28 significant terms were detected. Carbon metabolism (ko01200), glyoxylate and dicarboxylate metabolism (ko00630), glycine, serine, and threonine metabolism (ko00260), photosynthesis (ko00195), peroxisome (ko04146), photosynthesis—antenna proteins (ko00196), and starch and sucrose metabolism (ko00500) were significant categories during both development stages (Fig. S11).

Overall, GO and KEGG analyses results showed significant differences between T449 and E249 in the carbohydrate metabolic process during pollen development in T449 compared with E249, and DEGs were involved in sucrose synthase, glucose-6-phosphate1-dehydrogenase, 6-phosphofructokinase, and hexokinase. Sucrose is decomposed into monosaccharides by invertases and sucrose synthase, which are transported into cells and used for starch biosynthesis and other functions (Ruan et al. 2010; Ruan 2012). The sucrose synthase gene (*SUS3*) and hexokinase gene (*OsHXK1*) were significantly down-regulated at the meiosis stage. Two sucrose synthase genes (*SUS2* and *SUS3*) and three hexokinase genes (*OsHXK1, OsHXK3*, and *OsHXK7*) were up-regulated, and a hexokinase gene (*OsHXK10*) was found down-regulated at the single microspore stage (Table S11). Monosaccharides and sucrose transporters play a pivotal role in saccharides’ transportation from source to sink (Smeekens 2000; Wang et al. 2008). At the meiosis stage, a monosaccharide transporter (*OsMST6*) was found to be down-regulated. One sucrose transporter (*OsSUT5*) and two monosaccharide transporters (*OsMST1* and *OsMST8*) also exhibited down-regulation at the single microspore stage, and their expression patterns were confirmed by qRT-PCR (Table S11 and Fig. S4). These results indicated that saccharide transporters may cause a source–sink imbalance in T449 during pollen development.

### Determination of saccharide contents in anthers during meiotic and single microspore stages

**Fig. 4:**
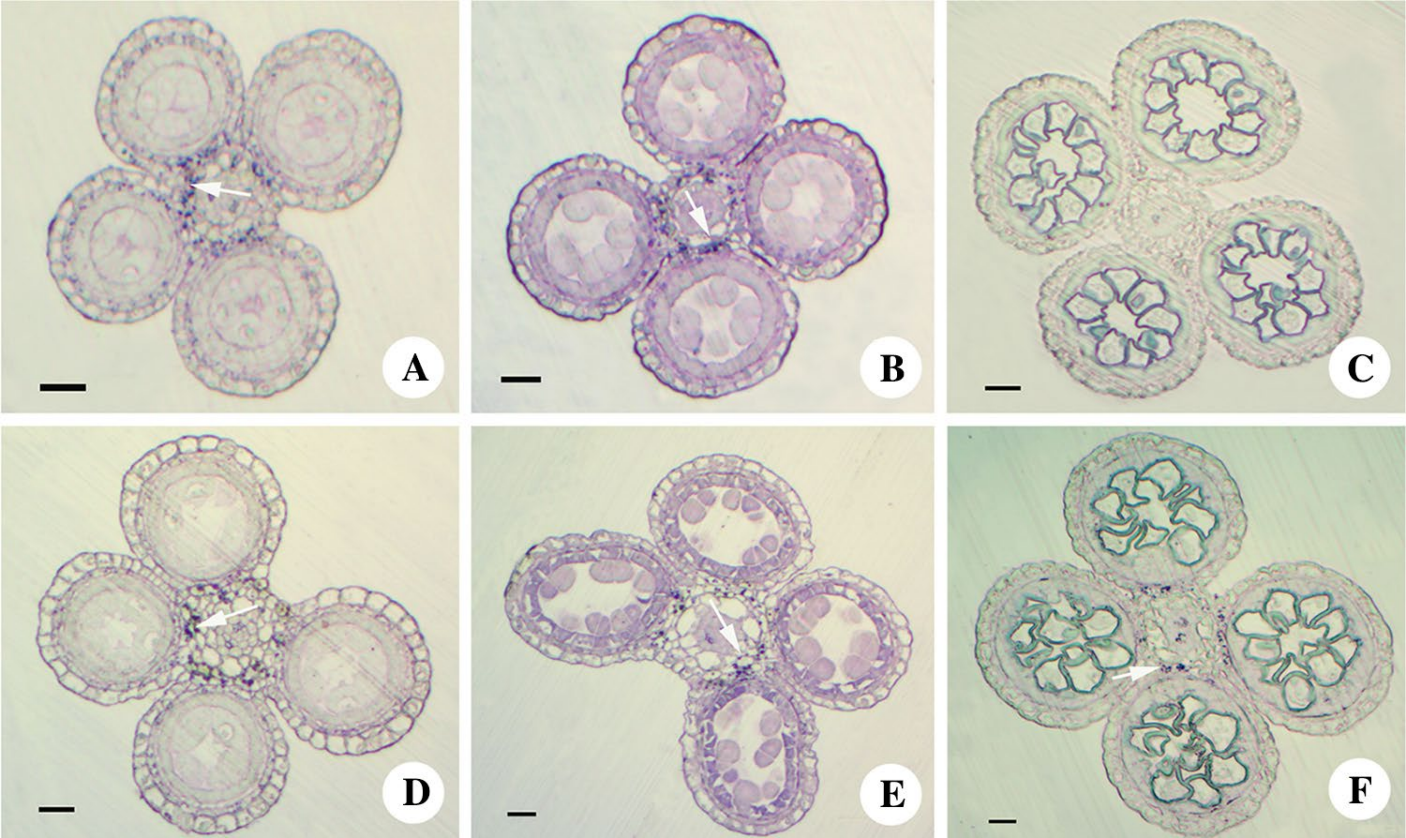
Comparison of saccharide distribution pattern in anthers of autotetraploid rice and its diploid counterpart. Anther cross sections of pre-meiotic stage (**a**), meiosis stage (**b**), and single microspore stage (**c**) in diploid rice. Anther cross sections of pre-meiotic stage (**d**), meiosis stage (**e**), and single microspore stage (**f**) in autotetraploid rice. Bar = 100 µm. Arrows indicate saccharides

**Fig. 5:**
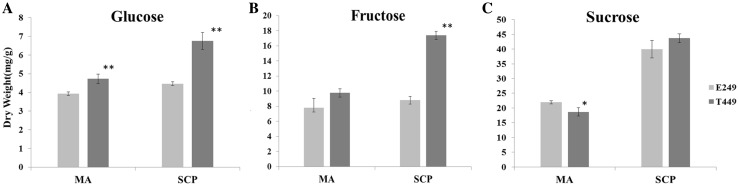
Saccharides’ contents in anthers of autotetraploid and diploid rice at meiotic and single microspore stages. **a** Glucose. **b** Fructose. **c** Sucrose. MA and SCP represent meiosis and single microspore stages, respectively. * and ** represent significant difference at *p* < 0.05 and *p* < 0.01, respectively. (Color figure online)

To verify our speculation that saccharides’ transporters disturb the source–sink relationship, the distribution of saccharides was investigated in T449 and E249 during pollen development. The results revealed that saccharides gathered at the connective region in E249 and T449 during the pre-meiotic and meiosis stages (Fig. 4). However, there was a significant difference in the distribution of saccharides between T449 and E249 during the single microspore stage (Fig. 4), and a large number of saccharides accumulated at the connective region in T449, but fewer saccharides were observed in E249 (Fig. 4). Moreover, we investigated the contents of fructose, glucose, and sucrose during pollen development. In the meiotic stage, the contents of glucose and sucrose significantly increased in T449, but there was no remarkable difference in contents of fructose between T449 and E249. At the single microspore stage, the contents of the fructose and glucose markedly increased in T449, but there was no obvious difference in the contents of sucrose between T449 and E249 (Fig. 5). These results indicated that monosaccharides (fructose and glucose) accumulated significantly in T449 during the single microspore stage.

### Meiosis-related and meiosis-specific genes showed down-regulation in autotetraploid rice

**Fig. 6:**
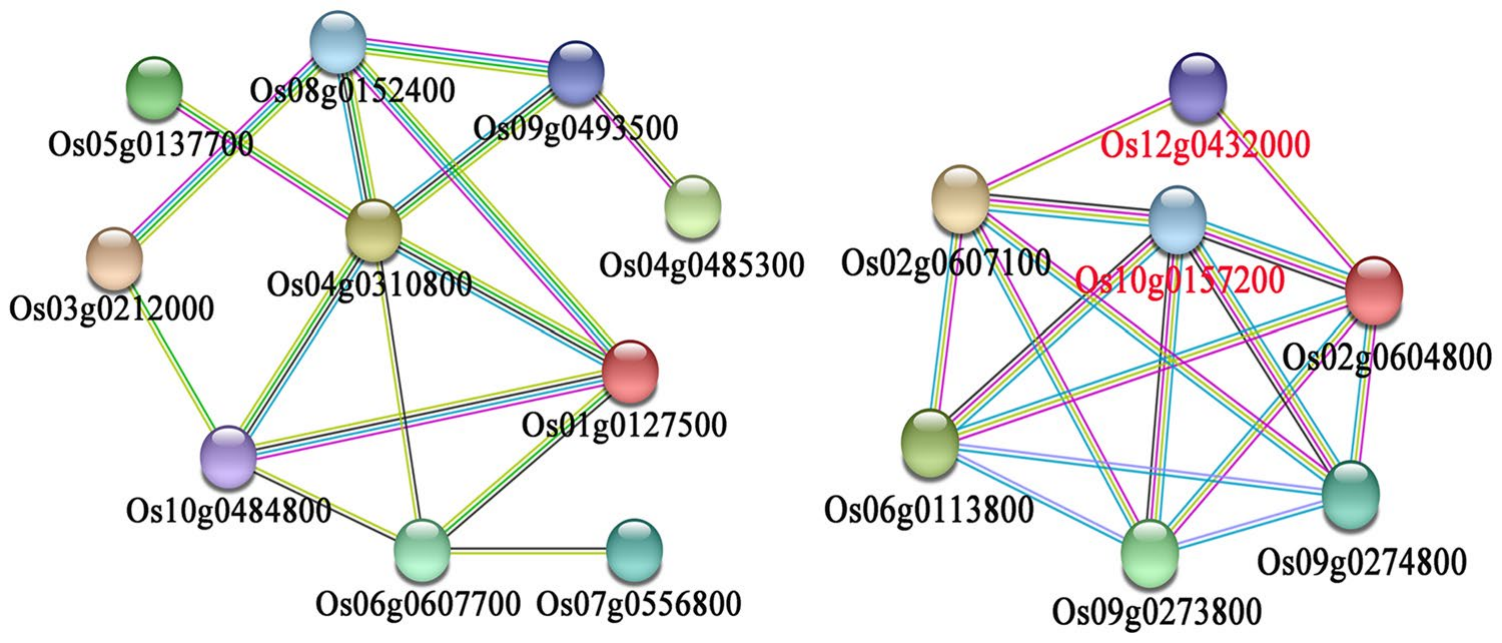
Predicted protein–protein interaction network of meiosis-specific (black) and meiosis-related (red) genes. Predicted protein–protein interaction subnetwork constructed using meiosis-specific and meiosis-related differently expressed genes between autotetraploid and diploid rice during meiosis. (Color figure online)

As comparative transcriptome profiling was used to analyze the DEGs during pollen mother cell (PMC) meiosis, we concentrated on meiosis-stage-specific and meiosis-related genes detected between T449 compared to E249 during the meiosis stage. We compared the DEGs to the transcriptomic data in rice or *Arabidopsis* meiosis-stage-specific genes and meiosis-related genes (Fujita et al. 2010; Tang et al. 2010; Deveshwar et al. 2011; Yant et al. 2013; Wright et al. 2015), and identified five meiosis-related and 70 meiosis-stage-specific genes (Table S12). These genes exhibited at least a twofold change in T449 compared to E249, including 9 up-regulated and 66 down-regulated genes. Interestingly, 60 genes were found to be down-regulated at the meiosis stage, but up-regulated at the single microspore stage, and only two genes showed the opposite tendency, i.e., up-regulated at the meiosis stage, but down-regulated at the single microspore stage. Thirteen of 75 specific genes displayed differential expression patterns at the meiosis stage, including 6 up-regulated and 7 down-regulated genes. Moreover, five meiosis-related genes were also detected, including one up-regulated and four down-regulated genes. The up-regulated meiosis-related (*Os05g0274200*) gene encodes DNA mismatch repair protein. Four meiosis-related genes, namely, topoisomerase B (*OsMTOPVIB, Os06g0708200*), F-box (*OsMOF, Os04g0464966*), cyclin-dependent kinase F-2 (*Os10g0157200*), and cyclin-dependent kinase G-1 (*Os12g0432000*), were found to be down-regulated, and their expression levels were verified by qRT-PCR (Fig. S4). We performed the predicted protein–protein interactions of 75 meiosis-stage-specific and meiosis-related genes using STRING v10. The results showed that 17 genes constituted 2 main genetic sub-networks (Fig. 6), and all exhibited down-regulation at the meiotic stage, including 2 meiosis-related genes and 15 meiosis-specific genes. The meiosis-related gene, *Os12g0432000*, encodes cyclin-dependent kinase G-1 and interacted with two meiosis-specific genes, including two cyclin genes (*Os02g0604800* and *Os02g0607100*). The meiosis-related gene, *Os10g0157200*, was annotated as cyclin-dependent kinase F-2, which interacted with five meiosis-specific genes, including two cyclin genes (*Os02g0604800* and *Os02g0607100*), two dimerisation domain containing genes (Skp1 family, *Os09g0273800* and *Os06g0113800*), and a SKP1-like gene (*Os09g0274800*).

## Discussion

### Whole-genome re-sequencing reveals that low fertility was not caused by SNPs’ and InDels’ variations between autotetraploid and diploid rice

Next-generation sequencing technologies have enabled re-sequencing of massive genomes, detection of DNA variations, and development of polymorphic molecular markers (Weigel and Mott 2009; Huang et al. 2012). The previous studies have reported the polymorphisms in genomic sequences of diploid rice, and discovered abundant genetic variations and important mutant genes (Jain et al. 2014; Fu et al. 2016). In the present study, a total of 82 SNPs and 182 InDels were detected in autotetraploid rice compared to diploid rice, and only three non-synonymous SNPs and six large-effect InDels were identified, which were associated with three and six genes/proteins, respectively. These genes/proteins were related to different categories, such as four disease-resistance genes (*Os11g0224900, Os01g0158600, Os11g0597700*, and *Os11g0686500*), cysteine protease family genes (*Os04g0402300*), methyltransferase (*Os09g0415700*), pectinesterase (*Os01g0634600*), and two expressed proteins (*Os08g0528700* and *Os10g0134033*, Table S4). This result suggests that the differences in pollen fertility between autotetraploid and diploid rice were not caused by SNP and InDel variations. Although 60 CNVs, which were associated with 617 genes, including two meiosis-related genes, were detected, the expression patterns of two meiosis-related genes exhibited non-significant differences between diploid and autotetraploid rice at the meiosis and single microspore stages by qRT-PCR. This result also revealed that CNV variation may not be the main reason for the low fertility of autotetraploid rice.

### Abrupt changes in meiosis-related genes and abnormal chromosome behaviour may lead to partial pollen sterility in autotetraploid rice

Meiosis process has a great effect on plant reproductive development (Luo et al. 2014). Autotetraploid rice has four sets of homologous chromosomes, and chromosome behaviour is more complicated in autotetraploid rice than in the diploid counterpart. Abnormal chromosome behaviour is a major reason for pollen abortion in autotetraploid rice (Luan et al. 2007, 2009; He et al. 2011a, b; Wu et al. 2014). There are many types of abnormal chromosome behaviour in the autotetraploid rice, including chromosome straggling and chromosome lagging (Wu et al. 2014). In the present work, high frequency of abnormal chromosome behaviour was found in autotetraploid rice, especially in anaphase II. The chromosome configurations have obvious difference between autotetraploid rice and diploid rice, and the frequencies of univalents and multivalents were higher in autotetraploid rice than diploid rice.

In the previous studies, a number of meiosis-related genes were detected in plants (Fujita et al. 2010; Yant et al. 2013; Luo et al. 2014; Wright et al. 2015), and more than 300 meiosis-specific genes were identified in rice anther (Fujita et al. 2010; Tang et al. 2010; Deveshwar et al. 2011). The down-regulation of 1 meiosis-related and 54 meiosis-stage-specific genes increased pollen sterility loci interactions in autotetraploid rice hybrids (Wu et al. 2015). In the present research, we detected 5 meiosis-related and 70 meiosis-specific genes that showed significant differential expression patterns between autotetraploid rice and its diploid counterpart. Four meiosis-related genes displayed down-regulation, and were involved in the formation of DNA double-stranded DNA breaks (DSBs), as well as synapsis and recombination during male meiosis. *OsMTOPVIB* (*Os06g0708200*) is a TopoVIB-like protein and essential for meiotic DSB formation (Xue et al. 2016). *OsMOF* (*Os04g0464966*) is an F-box gene, which was predominantly active during leptotene to pachytene of prophase I and participates in telomere bouquet formation, homologous chromosome pairing and synapsis, and DSB repair (He et al. 2016). The down-regulation of *OsMTOPVIB* and *OsMOF* may increase the number of univalents and trivalents at diakinesis and metaphase I in T449. *Os10g0157200* and *Os12g0432000* encoded cyclin-dependent kinase, which is homologue to *CDGK1* in *Arabidopsis thaliana. CDKG1* protein kinase is essential for synapsis and recombination during male meiosis in *Arabidopsis* (Zheng et al. 2014). The down-regulation of *CDKG1* may increase the percentage of chromosomal synapsis abnormalities in autotetraploid rice. The abrupt expression patterns of these meiosis-related and meiosis-specific genes may lead to abnormal chromosome behaviour, and cause low pollen fertility in autotetraploid rice.

### Expression changes in saccharides’ metabolism-related genes may contribute to saccharides’ abnormal distribution and cause partial pollen sterility in autotetraploid rice

The carbohydrate is the main nutrient in mature pollen that provides the necessary energy for pollen tube growth and pollen development. The meiotic and microspore stage are vital stages, which supply carbohydrates from the tapetum to microspores. This process involves the transport and degradation of polysaccharides, the formation and transport of monosaccharides, and the generation and degradation of starch (Lim et al. 2006; Kocal et al. 2008; Ruan et al. 2010). Sucrose transporters are important proteins for the translocation of sucrose from source to sink organs (Smeekens 2000). Lemoine et al. (1999) identified a pollen-specific sucrose transporter (*NtSUT3*) in tobacco, which participates in pollen development and supplies nutrition to pollen tubes. The deletion of the Os*SUT1* gene could damage pollen function in rice (Hirose et al. 2010). These studies demonstrated that the sucrose transporter plays an important role in plant pollen development. In our study, the sucrose transporter (*OsSUT5*) was down-regulated in T449 during the single microspore stage, which may affect the supply of sucrose during pollen development.

Sucrose invertase and sucrose synthase are the two key enzymes in sucrose metabolism in plants (Hirose et al. 2008; Ruan 2012). Here, RNA-seq data exhibited non-significant changes in the expressions of sucrose invertase genes. Sucrose synthase 3 (*OsSUS3*) was down-regulated at the meiotic stage and up-regulated at the single microspore stage, and *OsSUS2* was up-regulated at the single microspore stage. In addition, hexokinase genes have a great influence on the plant sugar signalling pathway (Cho et al. 2006, 2009; Kim et al. 2016). The hexokinase 10 (*OsHXK10*) plays a vital role in pollen germination, anther dehiscence, and hence grain filling in rice (Xu et al. 2008). Here, three hexokinase genes (*OsHXK1, OsHXK3*, and *OsHXK7*) were significantly up-regulated at the single microspore stage. The hexokinase genes, *OsHXK1* and *OsHXK10*, were found to be down-regulated at the meiotic stage and single microspore stage in autotetraploid rice compared to the diploid counterpart. We speculated that abrupt expressions of sucrose synthase and hexokinase genes may cause abnormal pollen development in autotetraploid rice.

Monosaccharide transporters play important roles in pollen development (Wang et al. 2008). Here, cross sections of autotetraploid rice anthers showed that a number of saccharides accumulated in the connective region during the single microspore stage, unlike diploid rice (Fig. 4). Moreover, the monosaccharide contents of autotetraploid rice were significantly higher than diploid rice during single microspore stage (Fig. 5). The expression of monosaccharide transporter 8 (*OsMST8*) has a pronounced effect on pollen development of rice (Mamun et al. 2006). The expression levels of *OsMST8* decreased significantly in the *cas* mutant, which reduce the sugar contents of flower organ and cause pollen sterility (Zhang et al. 2010). Similarly, *OsMST8* was found to be down-regulated during the single microspore stage in autotetraploid rice, which showed partial pollen sterility. These results indicated that abnormal accumulation of monosaccharides and the differential expressions of monosaccharide transporters may lead to partial pollen abortion in autotetraploid rice.

In conclusion, abnormal chromosomal behaviour and saccharides’ distribution were observed in autotetraploid rice, and many meiosis and carbohydrate metabolism-related genes displayed abrupt expression patterns during pollen development in autotetraploid rice. Therefore, we inferred that differential expression patterns of these genes may cause low pollen fertility in autotetraploid rice. Our results further the understanding of pollen development in autotetraploid rice, and revealed that carbohydrate metabolism and chromosomal behaviour abnormalities jointly cause partial pollen abortion in autotetraploid rice. Future studies will focus on the functional analysis of meiosis and saccharide metabolism-related genes, which were down-regulated in autotetraploid rice compared to diploid rice.

## Electronic supplementary material

Below is the link to the electronic supplementary material.

Supplementary material 1 (DOCX 29 KB)

Supplementary material 2 (DOCX 2413 KB)

Supplementary material 3 (DOCX 22 KB)

Supplementary material 4 (XLSX 845 KB)
