# Supplementary material for: Cytological and transcriptome analyses reveal abrupt gene expression for meiosis and saccharide metabolisms that associated with pollen abortion in autotetraploid rice
Source: Mol Genet Genomics. 2018 Jul 4;293(6):1407–20. doi: 10.1007/s00438-018-1471-0 (PMC6244853; doi:10.1007/s00438-018-1471-0)
Supplement: Supplementary file 1 — Supplementary material 1 (DOCX 29 KB) [file 438_2018_1471_MOESM1_ESM.docx]

Supplementary Material

**Cytological and transcriptome analyses reveal abrupt gene expression for meiosis and saccharide metabolism that associated with pollen abortion in autotetraploid rice**

**Journal: Molecular Genetics and Genomics**

Authors: Lin Chen^†^·Muhammad Qasim Shahid^†^·Jinwen Wu·Zhixiong Chen·Lan Wang·Xiangdong Liu*

*Corresponding author: Tel. and Fax: +86 20 85280205

* E-mail address: xdliu@scau.edu.cn (XDL)

**Additional files**

**Supplementary Fig. S1.** Chromosome behaviour during PMC meiosis in diploid rice (E249).

**Supplementary Fig. S2.** qRT-PCR expression profiles of *OsAM1* and *OsRAD17* during pollen development (meiosis (A) and single microspore stage (B)) using qRT-PCR in diploid (E249) and autotetraploid rice (T449).

**Supplementary Fig. S3.** Principal component analysis (PCA) of diploid (E249) and autotetraploid rice (T449).

**Supplementary Fig. S4.** Validation of DEGs in diploid (E249) and autotetraploid rice (T449) during pollen development.

**Supplementary Fig. S5.** Venn diagram of DEGs in diploid and autotetraploid during meiosis and single microspore stage.

**Supplementary Fig. S6.** Number of DEGs belonging to different transcription factor families detected in autotetraploid and diploid rice.

**Supplementary Fig. S7.** Significant GO terms of DEGs during the meiosis and single microspore stages (*p*-value < 0.05).

**Supplementary Fig. S8.** Significant GO terms of DEGs at the meiotic stage.

**Supplementary Fig. S9.** Significant GO terms of DEGs at the single microspore stage.

**Supplementary Fig. S10.** KEGG pathways enriched at the (A) meiosis and (B) single microspore stage.

**Supplementary Fig S11.** Significant ko terms of DEGs at both stages (*p*-value < 0.05). MA and SCP represent meiosis and single microspore stage.

**Supplementary Table S1.** Floret length during rice pollen development in autotetraploid (T449) and diploid (E249) rice.

**Supplementary Table S2.** Information of PCR primers used for identification of SNPs and InDels.

**Supplementary Table S3.** List of primers used for qRT-PCR.

**Supplementary Table S4.** List of DNA polymorphisms in T449 compared to E249.

**Supplementary Table S5.** Significant GO terms of genes related to CNVs in autotetraploid (T449) compared to diploid (E249) rice.

**Supplementary Table S6.** Quality of RNA sequencing data and information of reads aligned to Nipponbare reference genome.

**Supplementary Table S7.** Correlation analysis between all samples.

**Supplementary Table S8.** Differentially expressed genes at meiosis stage.

**Supplementary Table S9.** Differentially expresed genes at single microspore stage.

**Supplementary Table S10.** Differentially expressed transcription factors in autotetraploid (T449) and diploid (E249) during rice pollen development.

**Supplementary Table S11.** Expression of DEGs associated with saccharide metabolism during pollen development stages.

**Supplementary Table S12.** Meiosis related and stage-specific genes detected during meiosis in autotetraploid rice.
