# Supplementary material for: Cytological and transcriptome analyses reveal abrupt gene expression for meiosis and saccharide metabolisms that associated with pollen abortion in autotetraploid rice
Source: Mol Genet Genomics. 2018 Jul 4;293(6):1407–20. doi: 10.1007/s00438-018-1471-0 (PMC6244853; doi:10.1007/s00438-018-1471-0)
Supplement: Supplementary file 2 — Supplementary material 2 (DOCX 2413 KB) [file 438_2018_1471_MOESM2_ESM.docx]

Supplementary Material

**Cytological and transcriptome analyses reveal abrupt gene expression for** **meiosis and saccharide metabolism that associated with pollen abortion in autotetraploid rice**

Lin Chen^1,2,†^ • Muhammad Qasim Shahid^1,2,†^ • Jinwen Wu^2^ • Zhixiong Chen^2^ • Lan Wang^2^ • Xiangdong Liu^1,2,^*

^1^State Key Laboratory for Conservation and Utilization of Subtropical Agro-Bioresources, South China Agricultural University, Guangzhou 510642, China

^2^Guangdong Provincial Key Laboratory of Plant Molecular Breeding, South China Agricultural University, Guangzhou 510642, China

^†^These authors contributed equally to this work.

*Corresponding author: Tel. and Fax: +86 20 85280205

* E-mail address: xdliu@scau.edu.cn (XDL)

**Supplementary Figures**


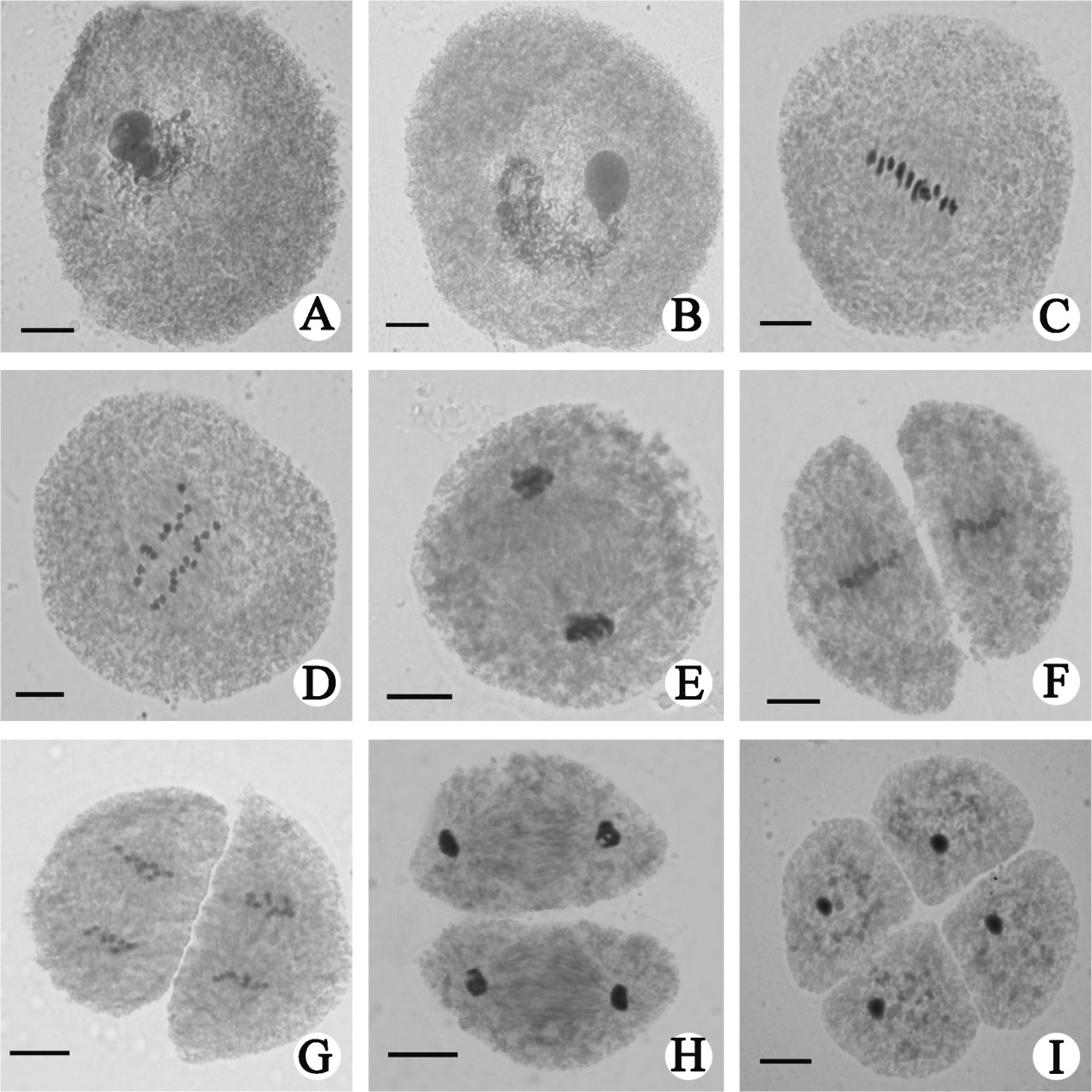


Supplementary Fig. S1. Chromosome behaviour during PMC meiosis in diploid rice (E249). A Zygotene，B Pachytene, C Metaphase I, D Anaphase I, E Telophase I, F Metaphase II, G Anaphase II, H Telophase II, I The tetrad stage. Bar=10μm.


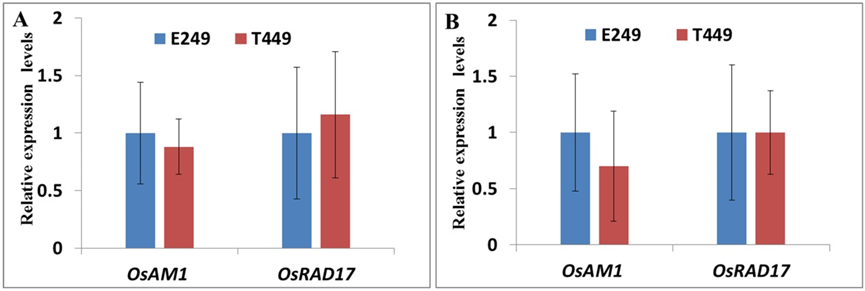


Fig. S2 qRT-PCR expression profiles of *OsAM1* and *OsRAD17* during pollen development (meiosis (A) and single microspore stage (B)) using qRT-PCR in diploid (E249) and autotetraploid rice (T449). The x- and y-axis represent the genes and relative expression levels, respectively.


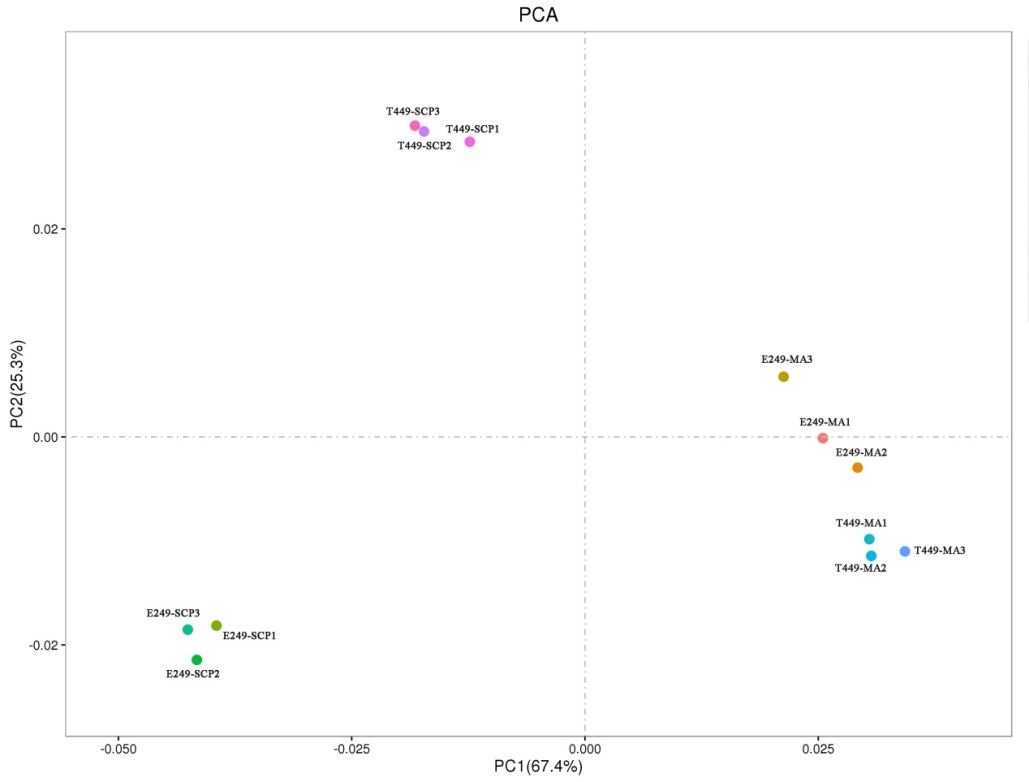


Supplementary Fig. S3. Principal component analysis (PCA) of diploid (E249) and autotetraploid rice (T449).


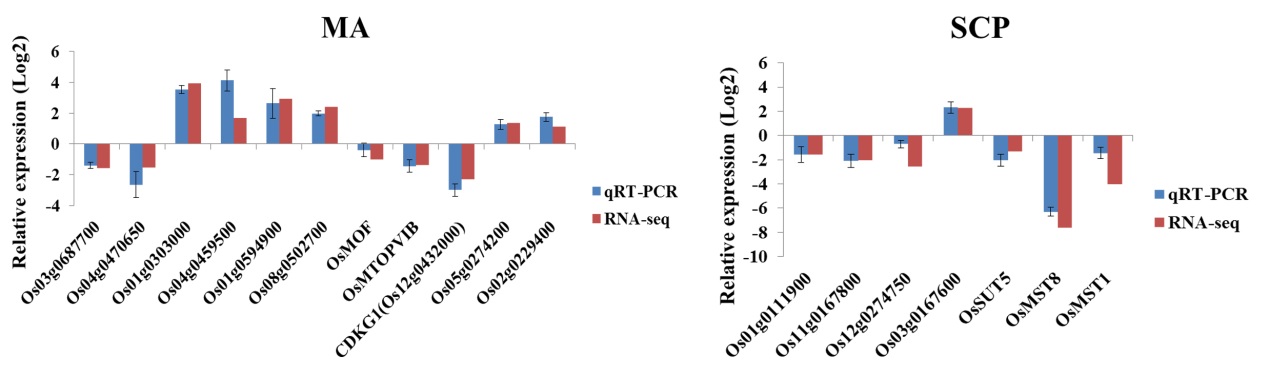


Supplementary Fig. S4. Validation of DEGs in diploid (E249) and autotetraploid rice (T449) during pollen development. MA and SCP represent meiosis and single microspore stage, respectively. The x- and y-axis are representing the genes and relative expression levels, respectively. The error bars indicate the standard deviation (SD) of three biological replicates.


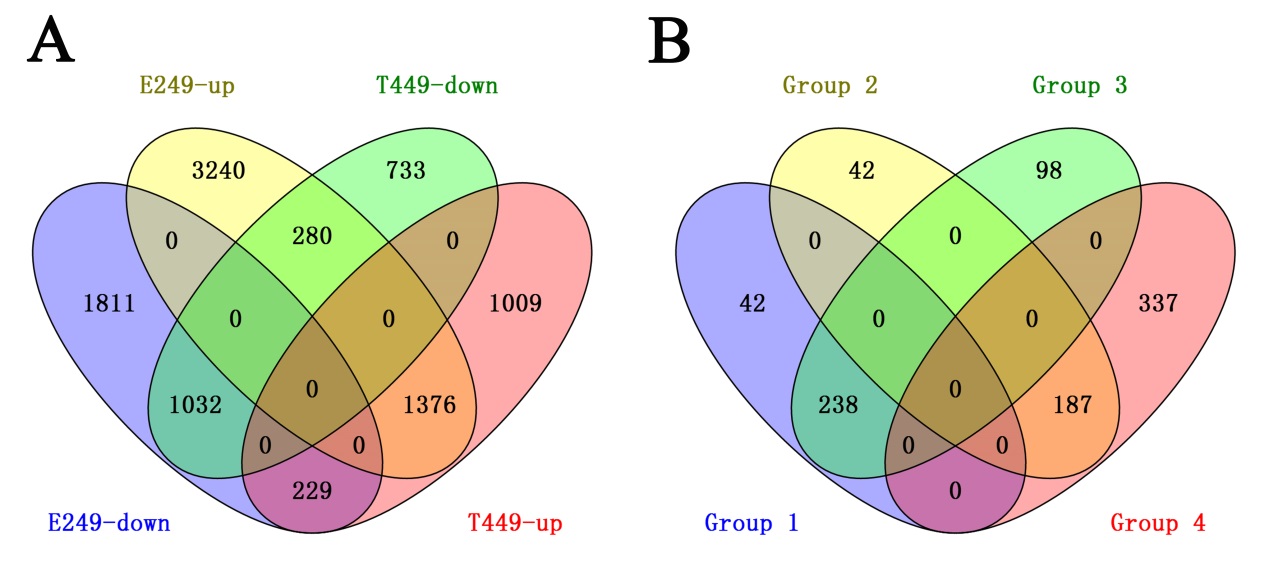


Supplementary Fig. S5. Venn diagram of DEGs in diploid and autotetraploid during meiosis and single microspore stage.

Venn diagram of DEGs during meiosis stage compared to single microspore stage in different ploidy rice (A). E249-down and E249-up represent the genes down-regulated and up-regulated in diploid rice (E249) during meiosis compared to single microspore stage, respectively. T449-down and T449-up represent the genes down-regulated and up-regulated in autotetraploid rice (T449) during meiosis compared to single microspore stage, respectively. Venn diagram of DEGs in different comparison groups (B). Group 1 represents the genes up-regulated in E249 but down-regulated in T449 during meiosis compared to single microspore stage, Group 2 represent genes down-regulated in E249 but up-regulated in T449 during meiosis compared to single microspore stage, Group 3 represent genes up-regulated at meiosis stage but down-regulated at single microspore stage in T449 compared to E249, Group 4 represent genes down-regulated at meiosis stage but up-regulated at single microspore stage in T449 compared to E249 during meiosis stage.


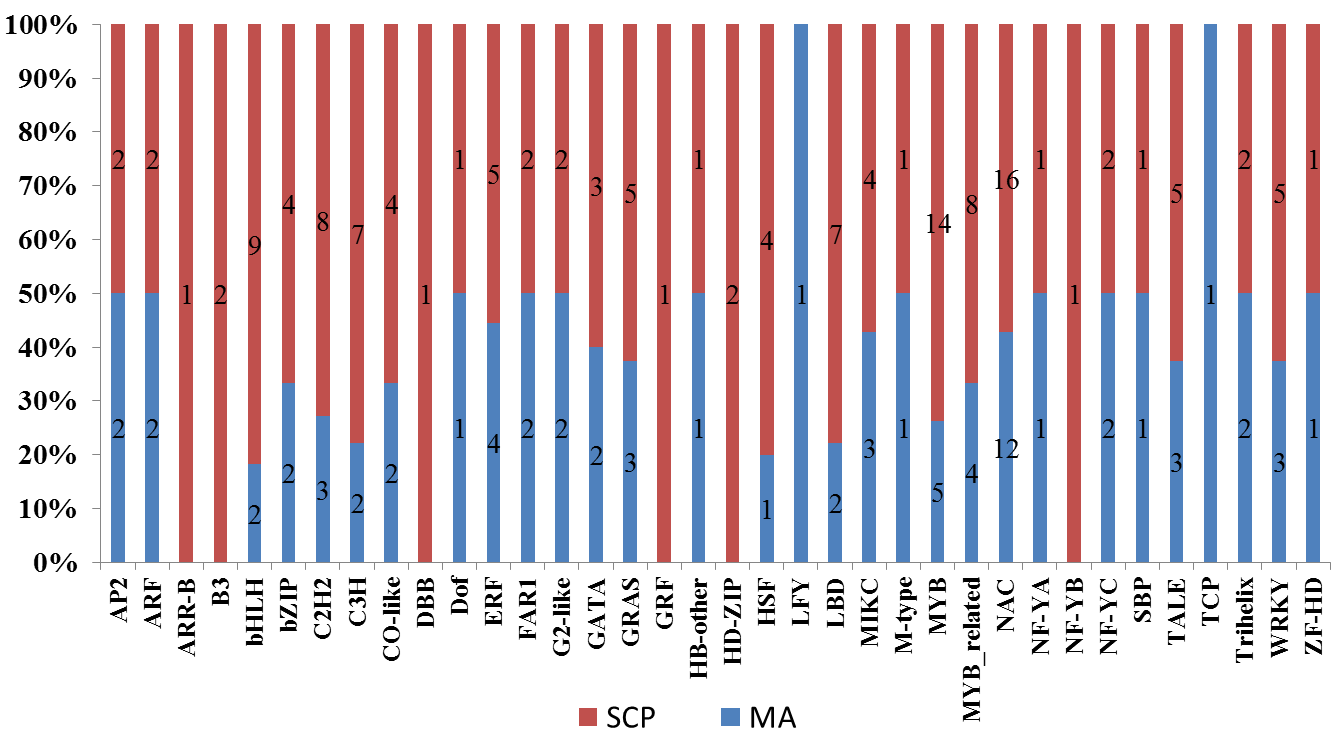


Supplementary Fig. S6. Number of DEGs belonging to different transcription factor families detected in the autotetraploid and diploid rice. In total, we identified 160 putative TF DEGs, which were classified into 36 families. MA and SCP represent meiosis and single microspore stage.


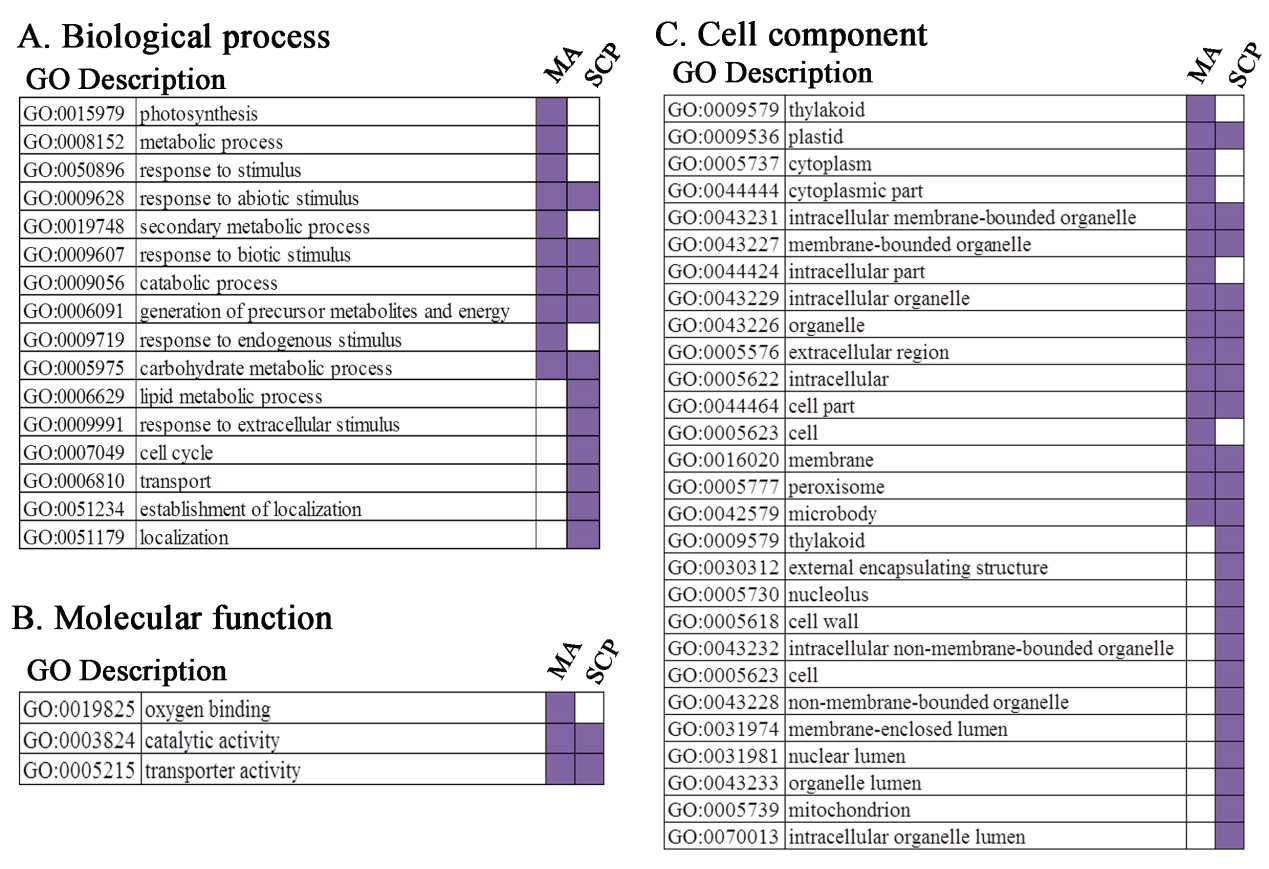


Supplementary Fig. S7. Significant GO terms of DEGs during the meiosis and single microspore stages (*p*-value < 0.05). A, Significant GO categories of biological process category in both stages. B, Significant GO categories of cell component category in both stages. C, Significant GO terms of molecular function category in both stages. MA and SCP represent meiosis and single microspore stage.


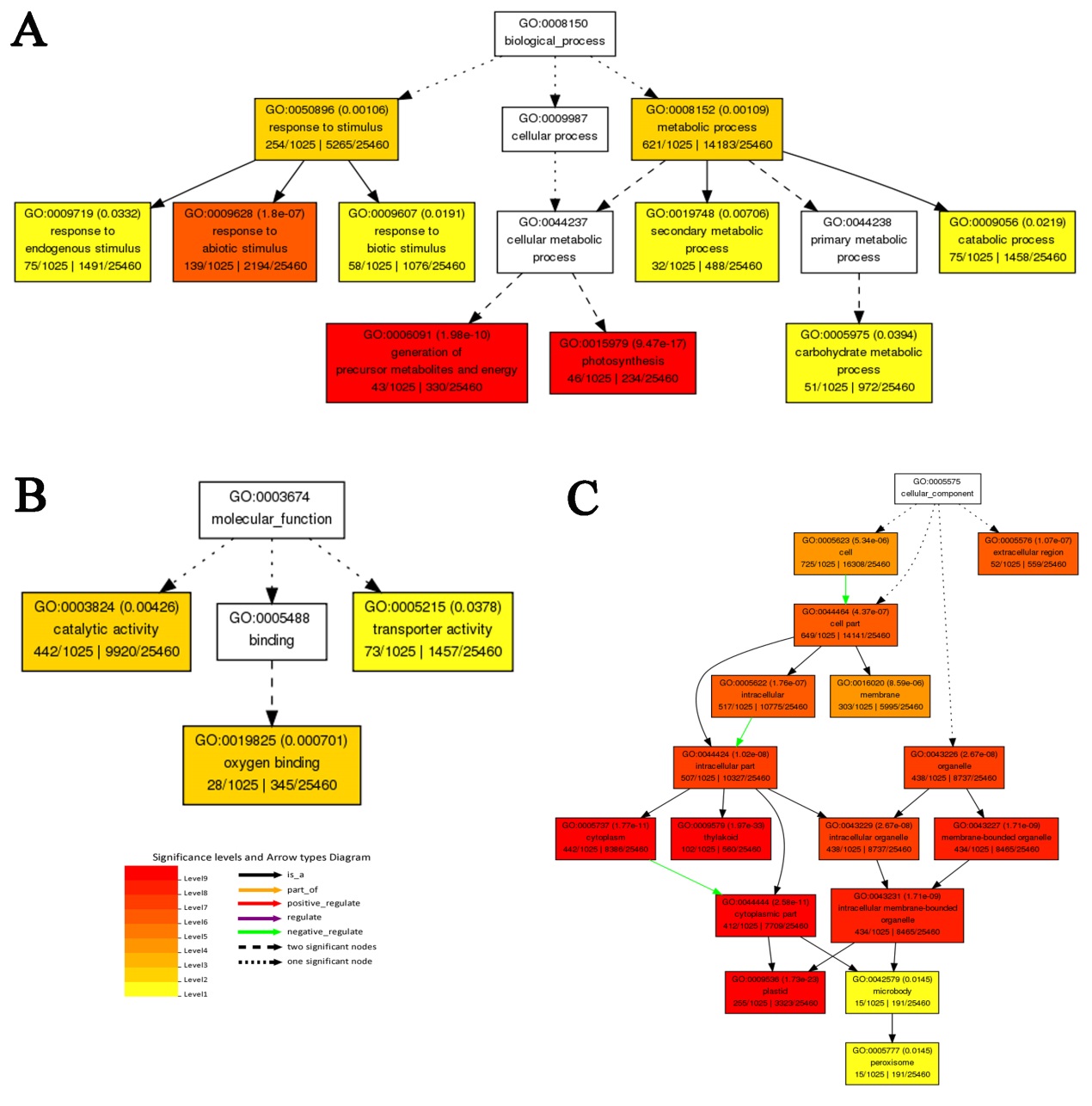


Supplementary Fig. S8. Significant GO terms of DEGs at the meiotic stage.

A, GO enrichment analysis of biological process category. B GO enrichment analysis of molecular function category. C, GO enrichment analysis of cellular component category.


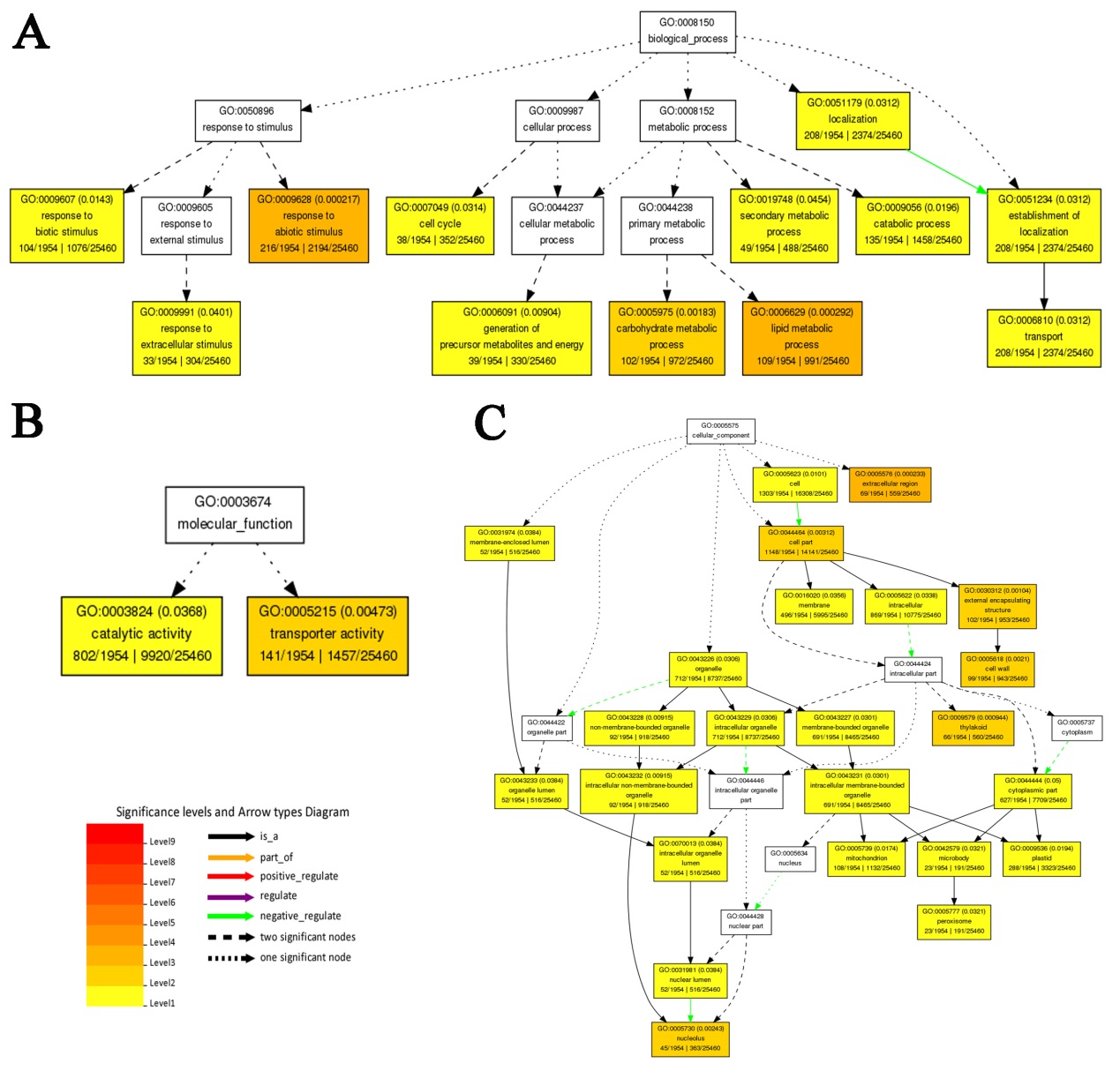


Supplementary Fig. S9. Significant GO terms of DEGs at the single microspore stage.

A, GO enrichment analysis of biological process category. B, GO enrichment analysis of molecular function category. C, GO enrichment analysis of cellular component category.


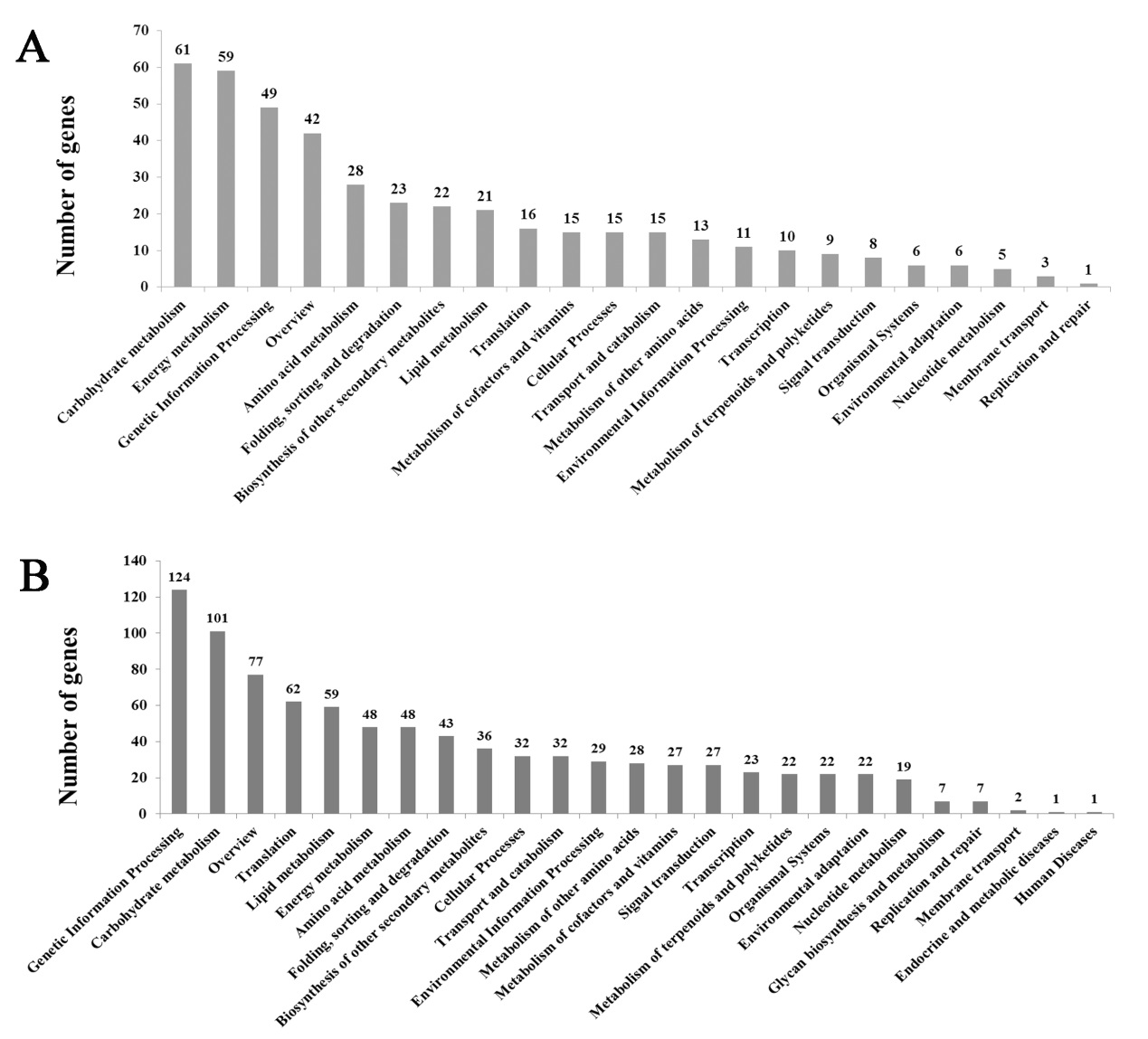


Supplementary Fig. S10. KEGG pathways enriched at the (A) meiosis and (B) single microspore stage.


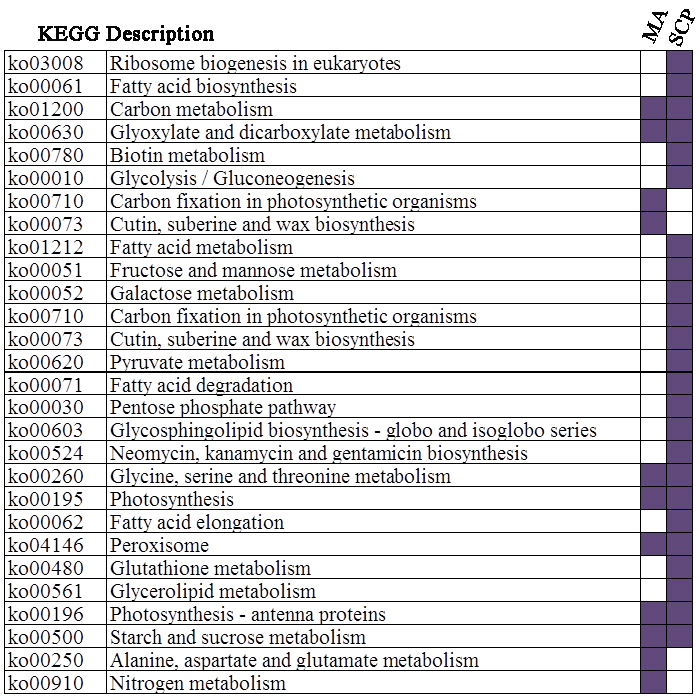


Supplementary Fig. S11. Significant ko terms of DEGs at both stages (*p*-value < 0.05). MA and SCP represent meiosis and single microspore stage.
