# Supplementary material for: Cytological and transcriptome analyses reveal abrupt gene expression for meiosis and saccharide metabolisms that associated with pollen abortion in autotetraploid rice
Source: Mol Genet Genomics. 2018 Jul 4;293(6):1407–20. doi: 10.1007/s00438-018-1471-0 (PMC6244853; doi:10.1007/s00438-018-1471-0)
Supplement: Supplementary file 3 — Supplementary material 3 (DOCX 22 KB) [file 438_2018_1471_MOESM3_ESM.docx]

Supplementary Table S1. Floret length during rice pollen development in autotetraploid (T449) and diploid (E249) rice.

| Materials | Meiotic stage (mm) | Single microspore stage (mm) |
| --- | --- | --- |
| E249 | 4.2-4.7 | 6-6.5 |
| T449 | 4.8-5.2 | 6.8-7.3 |

Supplementary Table S2. Information of PCR primers used for identification of SNPs and InDels.

| Name | Forward primer | Reverse primer | Amplification length (bp) |
| --- | --- | --- | --- |
| G-1 | AGTAGTCATGGAAGCCAGTT | GAAGGTCCAATTTGGCATGT | 549 |
| G-2 | CCGGCATTAAAATCAACACAG | CCATCACTATCCACCTCACT | 491 |
| G-3 | GGTAATGTAGTGTGTCGGTG | TCCTTTACCCCAGCTAACAA | 602 |
| G-4 | CAATTTGTTGATCGGCTTCG | AAACATGCTATACGTCCCCA | 470 |
| G-5 | GAGAGATGCGTCGTTTCATT | TCACTCAATATAAGTCGCACG | 450 |
| G-6 | AAGGCCACTACTCTACCATC | AACAGGATAGACCCCAATGT | 607 |
| G-7 | CCGACTTGTTCTTGTCTTTG | GAACTGATGTTGTTGAAGGC | 690 |
| G-8 | TCTGTGAGGCATTCGTGGTG | CTTAGGTGGCGGTATCAGGC | 782 |
| G-9 | CACATTCCCAAATTCATGCT | GGTTTACTATCCCACCGAAG | 592 |
| G-10 | TTGGATCGACCCTACCACTA | TTCATCTGTGCTTCGACTGT | 646 |
| G-11 | GCAACTTCAAACTACCGCC | CTCTTATTAGGGAAAGGGATGTAT | 552 |
| G-12 | CACTCAACCCAAGAACCAAT | ATGCAGTAGTATGTTGTGCG | 665 |
| G-13 | ATTAGTTAGTCGTTCGTGCG | GTTTCGTTATGCAGGGAGAA | 707 |
| G-14 | CAAGCAACGGATTTACTGGA | CCTAGAGATGTAGGCTACGG | 569 |
| G-15 | CCTTAGGATTTTGCTGGGAG | TTCGCGAAAGGAACATAGTC | 757 |
| G-16 | GCAAAGCCCACGAATCTAAC | TGCATCGAGGACACCACTATA | 698 |
| G-17 | GCTAGCAAAGCTAGATGGAC | GATCATCCCGAATCATTGGC | 573 |
| G-18 | GAGAGCAGGGAGATACAAGT | TAGTATAAAACCGGCGGAGT | 510 |
| G-19 | GCCACCCAATCCATAATAGC | GCTTGCTCTCCTTTATGTGAA | 494 |

Supplementary Table S3. List of primers used for qRT-PCR.

| Name | Forward primer | Reverse primer | Amplification length (bp) |
| --- | --- | --- | --- |
| *Os03g0687700* | CACGGGTGCTGTTTCTCTGA | CCATTCAGAAAGGGAACAACG | 100 |
| *Os04g0470650* | TGGTGAGGAGGTGGAGCATC | GAACCACTGGAACACGAAGC | 182 |
| *Os01g0111900* | CGAAAGCAGAAGCCAACCAT | GGAACTGTGTGCTCTCTCCTAAAG | 153 |
| *Os11g0167800* | GAGGGCGTCTACACGTCGG | CCGTGATCTTGTGCCTGTGC | 208 |
| *Os12g0274750* | GAAGCCGATGATACGGACGA | GTTCAGCAAGGTCGGATTCG | 181 |
| *Os02g0229400* | TTGTTGATGGCGATAAGGACC | TAAGTGGCACGCTCTGGTTC | 153 |
| *Os03g0167600* | TATGTGAACGGACAGAGGCAA | CCGTGTCTCCTGTGGTCAAA | 161 |
| *Os01g0303000* | CTGATTCTTACCATTATATGTTTGCT | CATTTCCCCCTTGAACTATCC | 138 |
| *Os04g0459500* | GTCGCCAACCAGTGGAAGTG | GGGGAAGAAAAGCAAAGAAAGA | 181 |
| *Os01g0594900* | CGGGCTCGACGATGACTACA | GCCGTGGTGGAAGCATTTAT | 175 |
| *Os08g0502700* | GTCATCGATATGTGCGTTGGA | GCCCATCTTGTAGAACTTGAGGTA | 202 |
| *Os04g0452700* (OsMST1) | TCATGACCGTCTTCATCGCC | AACCGCTTCCAGTACCAGTG | 100 |
| *Os01g0567500* (*OsMST8*) | CTTCAAGACCATCGGATTCG | CAGCGTCCCAAGAATGAACT | 178 |
| *Os02g0576600* (*OsSUT5*) | CGAAACTCCATCAAAGGCAC | CAACACAGCGAACGAATCAAC | 122 |
| *Os04g0464966* (OsMOF) | AAGGTTGACTTCTTGGTTGC | ATCCAATGTCACACTCGTTG | 169 |
| *Os06g0708200* (*OsMTOPVIB*) | CCTTGATTTACTGGGCGGTG | ACCATCCCATTTCTCCACGG | 203 |
| *Os12g0432000* (*CDKG1*) | ATGAAGTGCATCCGCTCGTA | CTGCACGATGTAGGGGTGG | 114 |
| *Os05g0274200* | TGCGGAAGAGAACAGCCAAT | CAGCGCAGAAAAACTGCCAT | 113 |
| ubiquitin | CAAGATGATCTGCCGCAAATGC | TTTAACCAGTCCATGAACCCG | 148 |
